# Smoking cessation increases levels of osteocalcin and uncarboxylated osteocalcin in human sera

**DOI:** 10.1038/s41598-020-73789-4

**Published:** 2020-10-08

**Authors:** Yasuhiro Kiyota, Hiroyasu Muramatsu, Yuiko Sato, Tami Kobayashi, Kana Miyamoto, Takuji Iwamoto, Morio Matsumoto, Masaya Nakamura, Hiroki Tateno, Kazuki Sato, Takeshi Miyamoto

**Affiliations:** 1 Department of Orthopedic Surgery, Keio University School of Medicine, 35 Shinano-machi, Shinjuku-ku, Tokyo, 160-8582 Japan; 2 Department of Advanced Therapy for Musculoskeletal Disorders II, Keio University School of Medicine, 35 Shinano-machi, Shinjuku-ku, Tokyo, 160-8582 Japan; 3 Department of Musculoskeletal Reconstruction and Regeneration Surgery, Keio University School of Medicine, 35 Shinano-machi, Shinjuku-ku, Tokyo, 160-8582 Japan; 4 Division of Pulmonary Medicine, Department of Medicine, Keio University School of Medicine, 35 Shinano-machi, Shinjuku-ku, Tokyo, 160-8582 Japan; 5 Institute for Integrated Sports Medicine, Keio University School of Medicine, 35 Shinano-machi, Shinjuku-ku, Tokyo, 160-8582 Japan; 6 Chuo Naika Clinic, 2-7-8 Nihon-bashi Ningyou-chou, Chuo-ku, Tokyo, 103-0013 Japan; 7 Department of Orthopedic Surgery, Kumamoto University, 1-1-1 Honjo, Chuo-ku, Kumamoto, 860-8556 Japan

**Keywords:** Metabolic disorders, Calcium and phosphate metabolic disorders, Osteoporosis

## Abstract

Smoking is thought to be a risk factor for osteoporosis development; however, the consequences of stopping smoking for bone homeostasis remain unknown. Here we conducted two separate human studies and show that bone mineral density was significantly lower in smokers than in non-smokers. The first was an observational study of pre- and post-menopausal healthy female smokers and non-smokers; the second included 139 current smokers determined to stop smoking. In the second study, levels of bone formation markers such as osteocalcin and uncarboxylated osteocalcin significantly increased after successful smoking cessation, as verified by significantly reduced levels of serum cotinine, a nicotine metabolite. Moreover, nicotine administration to mice reduced bone mineral density and significantly increased the number of osteoclasts in bone. Reduced bone mass phenotypes seen in nicotine-treated mice were significantly increased following nicotine withdrawal, an outcome accompanied by significantly reduced serum levels of tartrate-resistant acid phosphatase, a bone resorption marker. Taken together, our findings suggest that bone homeostasis is perturbed but can be rescued by smoking cessation.

## Introduction

Bone homeostasis is regulated by a delicate balance between osteoclastic bone resorption and osteoblastic bone formation; failure to maintain that balance promotes changes in bone mass^1^. Osteoporosis is a multifactorial disease caused by various factors including estrogen deficiency, aging, inflammatory or metabolic disease, or use of drugs such as steroids^2^. Lifestyle factors such as excessive alcohol consumption and smoking or lifestyle-related diseases such as diabetes mellitus are also risk factors for osteoporosis development^3,4^. Thus, although bone homeostasis is tightly regulated, it can be perturbed by several factors.

Numerous mechanisms underlie osteoporosis and may differ based on risk factors. Estrogen deficiency due to menopause results in accumulation of the transcription factor hypoxia inducible factor 1 alpha (HIF1α) in osteoclasts, in turn promoting osteoclast activation and bone loss^5^. Alcohol intake causes production of the alcohol metabolite acetaldehyde^6^, which acts as an oxidant and inhibits functions of osteoblasts and induces their apoptosis. Acetaldehyde-dependent oxidative stress can be reversed by treatment of osteoblasts with anti-oxidants^7^. A missense single nucleotide polymorphism in aldehyde dehydrogenase 2 (ALDH2), known as *rs671*, severely perturbs ALDH2 enzymatic activity, altering alcoholic metabolism and promoting alcohol flush syndrome^8,9^. Mice carrying *rs671* exhibit elevated serum acetaldehyde levels and significantly reduced bone mass relative to controls, even in the absence of alcohol consumption^10^. Furthermore, humans carrying *rs671* are at more significant risk for hip fracture and osteoporosis development, even without alcohol consumption, than are individuals who lack the mutation^7^.

Smoking is also a risk factor for fractures and osteoporosis development^11–13^. Smoking is reportedly associated with altered fracture healing resulting in delayed union seen in smokers compared to non-smokers^14^. Nicotine, which is taken in by smoking, disrupts bone homeostasis, and levels of the nicotine metabolite cotinine, are inversely correlated with bone mineral density (BMD)^15^. Nicotine acts via the alpha 7 nicotinic acetylcholine receptor (α7nAchR), a ligand-gated ion channel, which is permeable to Ca^2+^ and Na^+^ following acetylcholine or nicotine binding^16,17^. α7nAchR was demonstrated required for regulating levels of serum receptor activator of nuclear factor kappa B ligand (RANKL) and osteoprotegerin (OPG), which stimulate and inhibit, respectively, osteoclast differentiation^18^. We showed that α7nAchR-deficient mice exhibit significantly increased bone mass due to inhibition of osteoclastogenesis rather than to stimulation of osteoblastogenesis compared to wild-type mice^18^. However, it is still unclear whether smoking cessation has a positive effects on bones, and whether altered bone homeostasis can be reversed when individuals stop smoking.

In the current study, we demonstrate that: (1) in post-menopausal women, BMD is significantly lower in smokers than non-smokers; (2) serum levels of osteocalcin and uncarboxylated osteocalcin, both markers of bone formation markers, significantly increase after smoking cessation; (3) nicotine administration to wild-type mice reduces bone mass, a phenotype reversible by nicotine withdrawal; and (4) in mice treated as in (3) serum levels of tartrate resistant acid phosphatase 5b (TRAP5b), a marker of osteoclastic bone resorption, significantly decrease following nicotine withdrawal. Taken together, our results indicate that disturbance of bone homeostasis by smoking can be recovered by smoking cessation.

## Results

### Bone mineral density is significantly lower in smokers than in non-smokers among post-menopausal females

**Figure 1:**
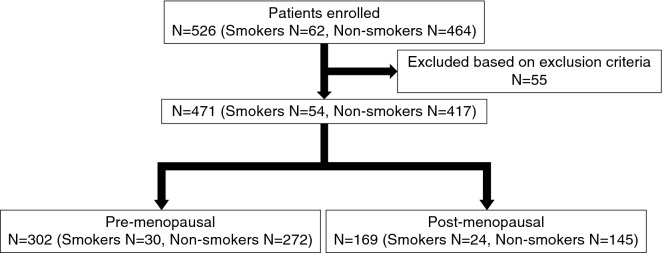
Flow chart of study subjects. Shown is a flow chart depicting groupings of indicated pre- and post-menopausal female volunteers, some smokers and others non-smokers. Analysis of these individuals is shown in Fig. 2.

**Figure 2:**
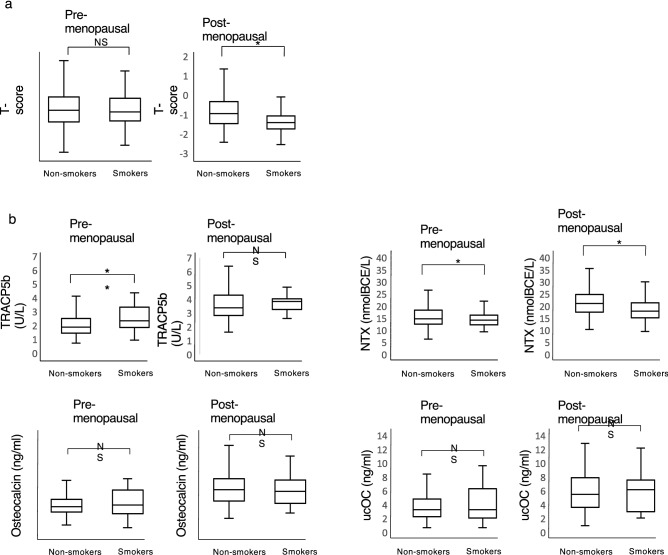
Bone mineral density and serum levels of markers of bone resorption or formation in pre- and post-menopausal subjects. (**a**) Among pre- and post-menopausal groups selected in Fig. 1, bone mineral density was analyzed as a T-score using an AOS-100 system in indicated subjects. (**b**) Serum levels of TRACP5b and NTX, both bone resorption markers, and the bone formation markers osteocalcin and ucOC were also analyzed in indicated subjects. Data represent mean T-score or serum levels of TRACP5b, NTX, osteocalcin or ucOC ± SD (*p < 0.05; **p < 0.01; NS, not significant).

For this analysis, we invited 526 women over 40 years of age (Fig. 1) and obtained informed consent from all. Of the 526 subjects, 62 and 464 were current smokers and non-smokers, respectively (Fig. 1). We excluded 55 subjects due to use of anti-osteoporosis or hormonal drugs, or due to pregnancy or lack of a complete data set (Fig. 1). Of the remaining 471 subjects, 54 and 417 were smokers and non-smokers, respectively (Fig. 1). Subjects were then subdivided into pre- (302) and post-menopausal (169) women (Fig. 1). Among pre-menopausal women, 30 and 272 were smokers and non-smokers, respectively, and 24 and 145 post-menopausal subjects were smokers and non-smokers, respectively (Fig. 1). The average age of smokers and non-smokers was comparable in both pre- and post-menopausal groups (Table 1). Basic characteristics of subjects, including body weight, height and BMI, were also similar between smokers and non-smokers in both pre- and post-menopausal women, although non-smokers were significantly taller than smokers in the pre-menopausal group (Table 1). Interestingly, bone mineral density (BMD) was significantly lower in smokers than in non-smokers among post-menopausal women (Fig. 2a). In pre-menopausal women serum levels of TRACP5b, a marker of osteoclastic bone-resorption, were significantly higher in smokers than in non-smokers (Fig. 2b). However, levels of serum N-terminal telopeptide of type 1 collagen (NTX), a different marker of bone resorption, were significantly lower in smokers than in non-smokers (Fig. 2b). Moreover, serum levels of osteocalcin and uncarboxylated osteocalcin (ucOC), both markers of bone formation, were comparable between smokers and non-smokers (Fig. 2b).

**Table 1:** Basic characteristics of pre- and post-menopausal subjects.

Pre-menopausal	Non-smoker (N = 272)	Smoker (N = 30)	*p* value
Age (years)	44.1 ± 4.5	44.8 ± 3.8	0.33
Body height (cm)	159.1 ± 5.3	156.8 ± 4.6	0.02
Body weight (kg)	54.3 ± 8.4	53.5 ± 9.9	0.68
BMI (kg/m^2^)	21.5 ± 3.2	21.7 ± 3.5	0.72

Post-menopausal	Non-smoker (N = 145)	Smoker (N = 24)	*p* value
Age (years)	55.2 ± 6.4	54.5 ± 4.1	0.48
Body height (cm)	156.4 ± 5.2	157.1 ± 5.1	0.53
Body weight (kg)	54.2 ± 8.9	52.2 ± 7.5	0.25
BMI (kg/m^2^)	22.2 ± 3.5	21.1 ± 2.7	0.11

### Smoking cessation elevates bone formation parameters in human subjects

**Figure 3:**
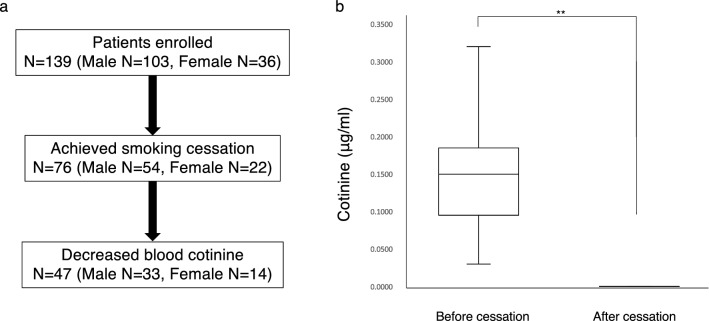
Blood cotinine levels decrease in subjects who successfully quit smoking. (**a**) Flow chart of subjects participating in a smoking cessation program. Informed consent was obtained from all 139 subjects enrolled, and their progression is shown as indicated. (**b**) Graph showing mean blood cotinine levels (μg/ml) ± SD monitored before and after smoking cessation (***p < 0.001).

For the second study, we invited 139 current smokers who had visited smoking cessation clinics as outpatients (Fig. 3). Among them, 103 and 36 were male and female, respectively (Fig. 3a). Among that 139, 54 males and 22 females reported success in stopping smoking (Fig. 3a). Among that 76, we performed blood tests in 64 subjects immediately before and then approximately 124 days after smoking cessation to evaluate cotinine levels (Fig. 3a). Blood cotinine levels significantly decreased after smoking cessation in 47 of 64 subjects (Fig. 3a,b). Based on this analysis, we concluded that these 47 subjects successfully quit smoking.

**Figure 4:**
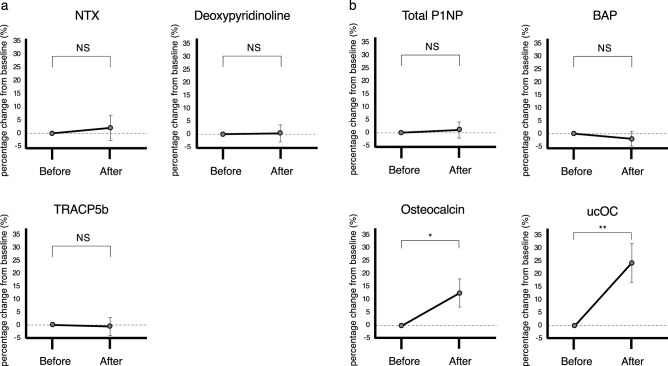
Changes in bone parameters after smoking cessation. (**a**) Analysis of bone-resorption parameters such as urinary NTX and deoxypyridinoline, and serum TRACP5b before (Before) and after (After) smoking cessation. Data are presented as mean percent changes from baseline values ± S.E after relative to before smoking cessation of NTX, deoxypyridinoline, and TRACP5b. (**b**) Analysis of bone formation markers evaluated in sera before (Before) and after (After) smoking cessation. Data represents mean percent changes from baseline values ± S.E after relative to before smoking cessation of total P1NP, BAP, osteocalcin and ucOC (*p < 0.05; **p < 0.01; NS, not significant). Dotted lines indicate baselines.

We then monitored changes in various biochemical parameters in individuals who had successfully stopped smoking (Fig. 4, Fig. S1 and Table 2). First, we focused on bone markers and found that parameters associated with bone resorption, namely, urinary NTX and deoxypyridinoline and serum TRACP5b levels, were unchanged after smoking cessation (Fig. 4a). However, serum levels of some parameters associated with bone formation, namely osteocalcin and ucOC, significantly increased after subjects quit smoking, although levels of other bone formation factors such as total type 1 procollagen-N-propeptide (P1NP) and bone specific alkaline phosphatase (BAP) did not (Fig. 4b). To identify potential regulators of osteocalcin and ucOC levels, we assessed other factors associated with bone metabolism (Table 2). However, serum levels of pentosidine, homocysteine, parathyroid hormone (PTH), 25(OH)D, and estradiol (E2), all implicated in regulating bone homeostasis, were comparable in these subjects before and after they stopped smoking (Table 2).

**Table 2:** Comparison of factors relevant to bone metabolism before and after smoking cessation.

	Before	After	*p* value
Pentosidine (μg/mg/CRE)	0.011 ± 0.0048	0.011 ± 0.0059	0.72
Homocysteine (nmol/mL)	11.3 ± 3.3	10.9 ± 3.2	0.54
PTH (pg/mL)	50.6 ± 29.7	51.2 ± 29.5	0.93
25(OH)D (ng/mL)	20.7 ± 6.5	21.3 ± 6.5	0.66
E2 (pg/mL)	40.1 ± 81.3	24.9 ± 35.4	0.25
Hgb A1c (%)	5.6 ± 0.55	5.7 ± 0.66	0.26

### Bone mass increases in mice after cessation of nicotine administration

**Figure 5:**
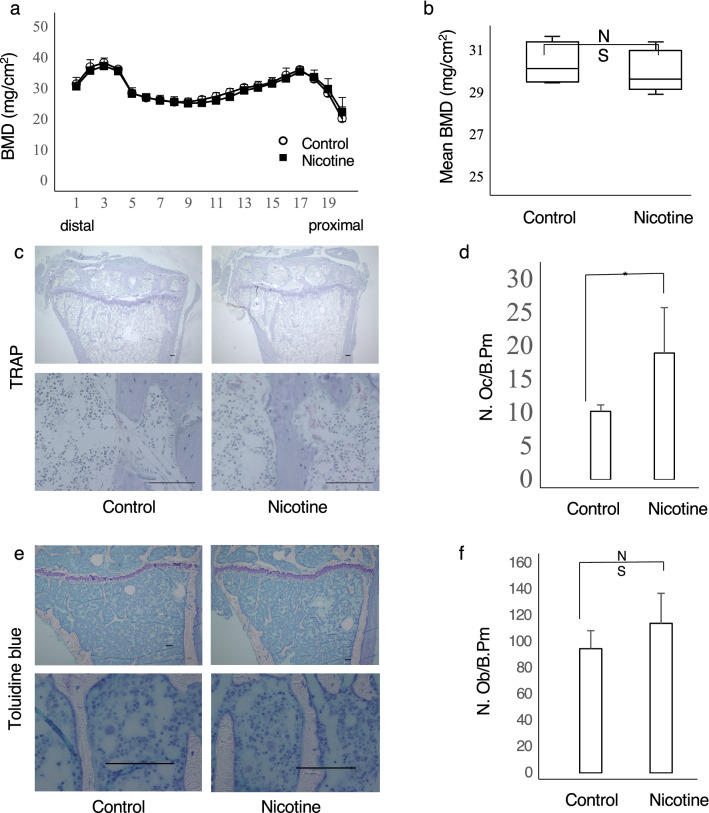
Nicotine administration stimulates osteoclastogenesis in mice. (**a**,**b**) Eight-week-old wild-type female mice were administered nicotine or PBS vehicle (control) for 3 weeks and then bone mineral density of femurs divided equally longitudinally was evaluated. Data represent mean BMD ± S.D. (NS, not significant; control, *n* = 4; nicotine, *n* = 5). (**c**–**f**) Eight-week-old wild-type female mice were administered nicotine or PBS vehicle (control) for 3 weeks and then tibial bone sections were stained with TRAP (**c**) or toluidine blue (**e**). The number of osteoclasts per bone perimeter (N.Oc/B.Pm) (**d**) or the number of osteoblasts per bone perimeter (N.Ob/B.Pm) (**f**) were then evaluated. Data represent mean N.Oc/B.Pm or N.Ob/B.Pm ± S.D (*p < 0.05; control, *n* = 4; nicotine, *n* = 5). Representative data of two independent experiments are shown. Bars = 100 μm.

**Figure 6:**
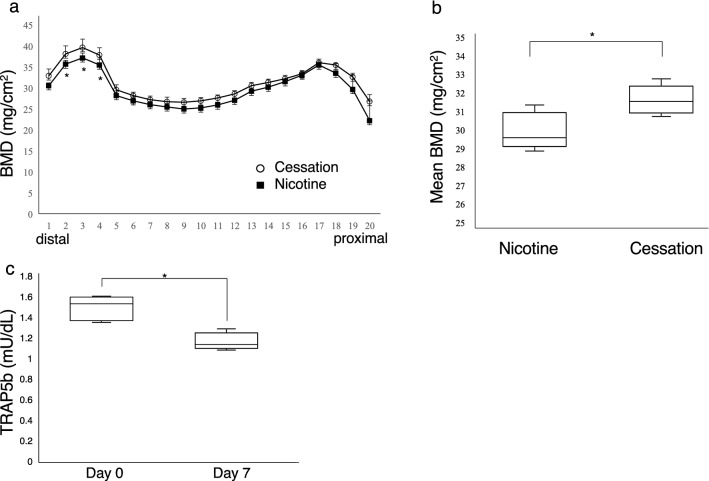
Bone mineral density increases following cessation of nicotine administration in mice. (**a**,**b**) Eight-week-old wild-type female mice were administered nicotine for 3 weeks. Then, nicotine administration was stopped and mice were maintained for two more weeks without nicotine administration. Bone mineral density of femurs divided equally longitudinally was evaluated serially from distal to proximal points in mice representing two time points: (1) after nicotine had been administered for 3 weeks (nicotine), and (2) 2 weeks after nicotine withdrawal (nicotine cessation). Mice in group 1 were 11 weeks old, and those in group 2 were 13 weeks old. Data represent mean BMD ± S.D. (*p < 0.05; nicotine, *n* = 6; nicotine cessation, *n* = 5). (**c**) Eight-week-old wild-type female mice were administered nicotine for 3 weeks. Then, nicotine administration was stopped and mice were maintained for seven more days without nicotine administration. Serum TRAP5b levels were evaluated by ELISA after nicotine had been administered for 3 weeks (day 0), and 7 days after nicotine withdrawal (day 7). Data represent mean serum TRACP5b (mU/dl) ± S.D. (*p < 0.05; *n* = 5).

Finally, we asked whether bone homeostasis would improve in nicotine-treated mice once drug was withdrawn (Fig. 5). To do so, first we conducted bone mineral density analysis, and TRAP and toluidine blue staining of femoral bone and tibial bone sections, respectively, in mice that had been treated with and without nicotine cross sectionally (Fig. 5). These data indicated that daily subcutaneous administration of nicotine to wild-type mice over a 3-week period starting at 8 weeks of age reduced bone mass relative to untreated controls, but those changes were not significant (Fig. 5a,b). However, when we examined bone sections from these mice, nicotine-administered mice showed a significant increase in the number of osteoclasts relative to controls (Fig. 5c,d), while osteoblast number was comparable in nicotine- and PBS-treated mice (Fig. 5e,f). We then examined bone mass longitudinally in nicotine-administered mice 2 weeks after nicotine withdrawal, when mice were 13 weeks of age (Fig. 6). These mice showed significantly elevated bone mineral density compared to mice evaluated immediately after 3 weeks of nicotine treatment (Fig. 6a,b). Twelve-week-old mice that had undergone 1 week of nicotine withdrawal also showed decreased levels of serum TRACP5b than did control mice treated with nicotine for 3 weeks (Fig. 6c).

## Discussion

Osteoporosis is a multifactorial disease brought on by various risk factors or their combination^2–4^. Among them, aging, menopause and genetic factors cannot be controlled as a means to antagonize osteoporosis development; however, lifestyle-related factors such as excessive alcohol consumption and smoking are manageable. In this study, we addressed effects of smoking and smoking cessation in two human studies. In the first, we assessed BMD differences in female pre- and post-menopausal smokers versus comparable non-smokers. That analysis revealed BMD to be significantly lower in post-menopausal smokers compared to non-smokers. In a second study, we evaluated male and female smokers before and after they quit smoking and found that levels of bone formation markers significantly increased within 124 days of smoking cessation. This analysis indicates that the negative effects of smoking on bone homeostasis can be reversed within a short period following smoking cessation, conferring a significant benefit for bone health. Also, mouse studies reported here show that BMD increases within a short period after nicotine discontinuation accompanied by significantly reduced serum levels of TRAP5b, a marker of bone resorption. Smoking is a well-known risk for several diseases, including osteoporosis, lung cancer, chronic obstructive pulmonary disease (COPD), and COVID-19^19^; our analysis adds to the body of evidence demonstrating that smoking cessation has positive effects on individuals’ health.

Nicotine promotes various effects in smokers. Among them, euphoria and decreased heart rate are due to vagal nerve stimulation. Nicotine intake reportedly promotes peripheral vessel vasoconstriction, which is associated with osteoporosis development or delayed fracture healing^20^. In mice, nicotine administration was demonstrated to elevate RANKL and to decrease OPG levels in sera to stimulate osteoclastogenesis^18^. Mice deficient in the nicotinic acetylcholine receptor α7nAchR exhibit a significantly decreased RANKL/OPG ratio and osteoclast number relative to controls and a significant increase in bone mass^18^. In this study, we demonstrated that interrupting administration of nicotine to mice increased bone mass and decreased levels of the bone resorption marker TRACP5b. We also observed increased levels of some markers of bone formation in humans who had stopped smoking. Thus, although we currently do not understand the reasons for differences in effects of smoking cessation on bone metabolism between humans and mice, it is clear that stopping smoking is a way to decrease the risk of osteoporosis development.

At present, we do not know precisely why only osteocalcin and ucOC levels changed after smoking cessation in human subjects. Nonetheless, these findings suggest that osteocalcin and ucOC respond more rapidly to smoking cessation than TRACP5b, making them potentially more appropriate markers of bone effects of smoking cessation. The osteocalcin-ucOC axis reportedly plays a role in regulating glucose metabolism^21^; however, since hemoglobin A1c (HgbA1c) levels were unchanged after smoking cessation (Table 2), we do not attribute changes in ucOC to improved glycemic control.

Relevant to study limitations, we did not collect BMD data from subjects who had stopped smoking, as most stopped smoking within 124 days, a period too short to observe significant changes in BMD. We evaluated smoking habits as either “Yes” or “No”, and did not inquire about amounts of smoking. Also, samples were collected at fasting but at various times of day. We note, however, that TRACP5b levels are reportedly unaffected by circadian rhythm and feeding^22^. Also, we did not follow up with subjects after successful smoking cessation, and samples were not collected before or after 124 days. Finally, the number of non-smokers evaluated exceeded the number of smokers: the proportion of smokers in our subjects was 11.5%, which is lower than global averages^23^. Nonetheless, we feel that conclusions relevant to reported changes in bone density are valid and that our study makes a significant contribution to the field.

In summary, we conclude that based on our findings, deleterious changes in bone homeostasis associated with smoking can be rescued by smoking cessation. Such changes in smoking behaviors should be encouraged as a potential means to improve bone metabolism and reduce risk of osteoporosis development.

## Materials and methods

### Human subjects

This study protocol was approved by an ethics committee at Keio University School of Medicine and carried out in accordance with guidelines of that committee. Informed consent was taken from all subjects prior to the study. We conducted two separate human studies. In the first, we invited 571 female medical workers in our university hospital, aged 39 to 64 years of age, and obtained informed consent from 526. All completed a self-reported questionnaire regarding current and/or previous menstruation status, smoking habits, past history of disease or drug usage. Based on that questionnaire, we excluded 55 due to medical complications and/or use of anti-osteoporosis or hormonal drugs, which are known to alter bone metabolism, or due to pregnancy or lack of a complete data set. The remaining 471 were divided into pre-menopausal (n = 302) and post-menopausal (n = 169) women, and in each group, smokers and non-smokers were assessed for bone mineral density (BMD) and serum bone markers.

The second study included 139 current smokers who intended to stop smoking. Of them, 76 reported they had stopped smoking, and 47 of those were blood cotinine-negative. In that group of 47 subjects, we evaluated levels of bone markers and other parameters in sera before and after smoking cessation.

### Measurements

In the first study, body height, weight, and serum levels of tartrate resistant acid phosphatase 5b (TRACP5b), N-terminal telopeptide of type 1 collagen (NTX), osteocalcin and undercarboxylated osteocalcin (ucOC) were assessed in all subjects, and body mass index (BMI) was calculated based on body height and weight data. TRACP5b (Nittobo, Fukushima, Japan) and NTX (Abbott Diagnostics Medical Co., Ltd, Tokyo, Japan) were analyzed by enzyme immunoassay (EIA) and enzyme-linked immunosorbent assay (ELISA), respectively. Osteocalcin (Roche, Basel, Switzerland) and ucOC (Sekisui Medical, Tokyo, Japan) were analyzed by an electrochemiluminescent immunoassay (ECLIA). BMD was analyzed using an AOS-100 system (Aloka, Tokyo, Japan), as described^24^.

In the second study, parameters associated with bone resorption, namely, urinary NTX and deoxypyridinoline and serum TRACP5b were assessed in all subjects as well as bone formation parameters including serum osteocalcin, uncarboxylated osteocalcin (ucOC), total type 1 procollagen-N-propeptide (P1NP), and bone specific alkaline phosphatase (BAP), as were pentosidine, homocystein, intact parathyroid hormone (PTH), 25(OH)VitD, estradiol (E2), and cotinine levels. TRACP5b (Nittobo, Fukushima, Japan), pentosidine (FSK, Kagawa, Japan) and deoxypyridinoline (SB Bioscience Co., Ltd, Osaka, Japan) were analyzed by EIA. PTH (Roche Diagnostics, Tokyo, Japan), osteocalcin (Roche), total P1NP (Roche), E2 (Roche), and ucOC (Sekisui Medical, Tokyo, Japan) were analyzed by ECLIA. BAP (Beckman Coulter, Pasadena, CA) and uNTX (Alere Medical Co., Ltd, Tokyo, Japan) were analyzed by radioimmunoassay. Homocysteine (YMC CO., LTD, Kyoto, Japan) was analyzed by high performance liquid chromatography (HPLC).

### Mice

C57BL/6J wild-type mice were purchased from Sankyo Labo Service (Tokyo, Japan). Eight-week-old wild-type female mice were randomly assigned to each treatment group. Nicotine (at a dosage of 84 μg/day) or PBS were injected subcutaneously every day. In the first examination, mice were administered nicotine or PBS vehicle (control) for 3 weeks. We then evaluated bone mineral density of femurs divided equally longitudinally. Tibial bone sections were stained with TRAP or toluidine blue, and the number of osteoclasts per bone perimeter (N.Oc/B.Pm) and the number of osteoblasts per bone perimeter (N.Ob/B.Pm) were then evaluated (control, *n* = 4; nicotine, *n* = 5).

In the second examination, mice were administered nicotine for 3 weeks. Then, nicotine administration was stopped, and mice were maintained for two more weeks without nicotine administration. We then evaluated bone mineral density of femurs divided equally longitudinally serially from distal to proximal points in mice representing two time points: (1) after nicotine had been administered for 3 weeks (nicotine), and (2) 2 weeks after nicotine withdrawal (nicotine cessation) (nicotine, *n* = 6; nicotine cessation, *n* = 5). In the third examination, mice were administered nicotine for 3 weeks. Nicotine administration was then stopped, and mice were maintained seven more days without drug. Serum TRAP5b levels were evaluated by ELISA after nicotine had been administered for 3 weeks (day 0), and 7 days after nicotine withdrawal (day 7) (*n* = 5).

For BMD analysis, femurs were removed, fixed in 70% ethanol, and subjected to dual energy x-ray absorptiometric (DEXA) scanning to measure BMD (mg/cm^2^) at proximal to distal points using a DCS-600R system (Aloka, Co. Ltd, Tokyo, Japan). Bone morphometric analysis and tartrate-resistant acid phosphatase (TRAP) staining were performed in tibiae, as described^25^.

All animals were maintained under specific pathogen-free conditions in animal facilities certified by the Keio University animal care committee. Animal protocols were approved by that committee and carried out in accordance with the committee’s guidelines. Animals were housed up to 6 mice per cage and kept on a 12 h light/ dark cycle. Water and food were available ad libitum. All animal studies were performed in accordance with the guidelines of the Keio University animal care committee, as described^26^.

### Serum TRAP5b assay in mice

Eight-week-old wild-type female mice were subcutaneously injected nicotine (at a dosage of 84 μg/day) every day for 3 weeks, and then nicotine administration was stopped. Sera were collected from nicotine-administered mice before and 7 days after nicotine cessation. TRAP5b levels in sera were assessed by ELISA, according to the manufacturer’s protocol (Immunodiagnostic Systems Limited, Boldon, Tyne & Wear, UK).

### Statistical analysis

Statistical analysis was performed using a non-parametric test (*p < 0.05; **p < 0.01; ***p < 0.001; NS, not significant, throughout the paper).

## Supplementary information

Supplementary Information.
