# Supplementary material for: Smoking cessation increases levels of osteocalcin and uncarboxylated osteocalcin in human sera
Source: Sci Rep. 2020 Oct 8;10:16845. doi: 10.1038/s41598-020-73789-4 (PMC7546721; doi:10.1038/s41598-020-73789-4)
Supplement: Supplementary file 1 — Supplementary Information. [file 41598_2020_73789_MOESM1_ESM.pdf]

Smoking cessation increases levels of osteocalcin and uncarboxylated osteocalcin in human sera

Yasuhiro Kiyota[a], Hiroyasu Muramatsu[f], Yuiko Sato[a,b,c], Tami Kobayashi[a,b,c], Kana Miyamoto[a,g], Takuji Iwamoto[a], Morio Matsumoto[a], Masaya Nakamura[a], Hiroki Tateno[d], Kazuki Sato[e] and Takeshi Miyamoto[a, b, c, g]

[a]Department of Orthopedic Surgery, [b]Department of Advanced Therapy for Musculoskeletal Disorders II, [c]Department of Musculoskeletal Reconstruction and Regeneration Surgery, [d]Division of Pulmonary Medicine, Department of Medicine, [e]Institute for Integrated Sports Medicine, Keio University School of Medicine, 35 Shinano-machi, Shinjuku-ku, Tokyo 160-8582, Japan, [f]Chuo Naika Clinic, 2-7-8 Nihon-bashi Ningyou-chou, Chuo-ku, Tokyo 103-0013, Japan, [g]Department of Orthopedic Surgery, Kumamoto University, 1-1-1 Honjo, Chuo-ku, Kumamoto 860-8556, Japan

Supplementary Figure 1. Changes in bone parameters after smoking cessation.

(a) Analysis of indicated bone resorption parameters Before and After smoking cessation. Data are presented as mean values ± S.E of each. (b) Analysis of indicated bone formation markers evaluated in sera Before and After smoking cessation. Data represents mean values ± S.E of each (*p < 0.05; **p < 0.01; NS, not significant).

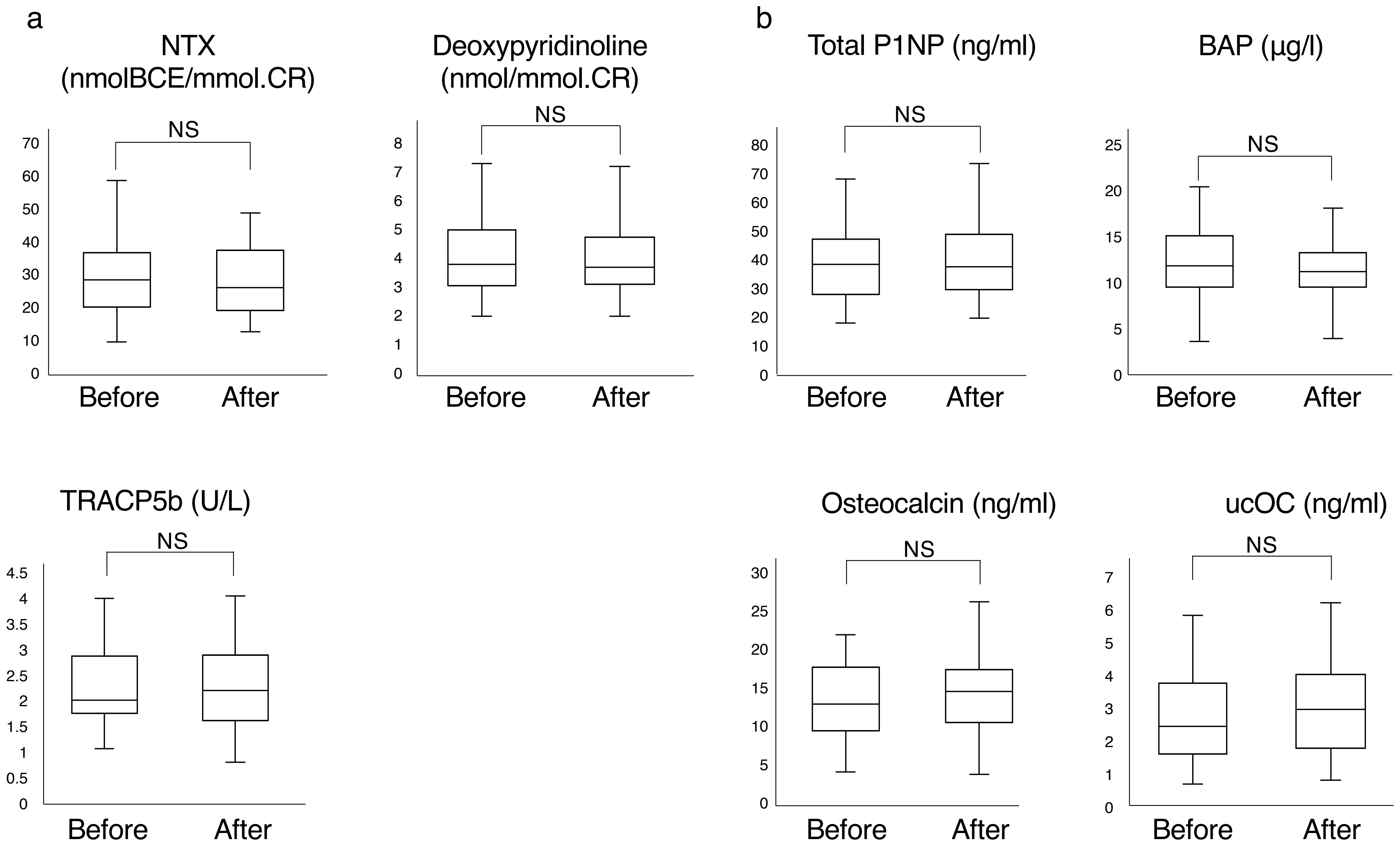
a
NTX
(nmolBCE/mmol.CR)
NS
0
10
20
30
40
50
60
70
Before
After
Deoxypyridinoline
(nmol/mmol.CR)
NS
0
1
2
3
4
5
6
7
8
Before
After
TRACP5b (U/L)
NS
0
0.5
1
1.5
2
2.5
3
3.5
4
4.5
Before
After
b
Total P1NP (ng/ml)
NS
0
10
20
30
40
50
60
70
80
Before
After
BAP (µg/l)
NS
0
5
10
15
20
25
Before
After
Osteocalcin (ng/ml)
NS
0
5
10
15
20
25
30
Before
After
ucOC (ng/ml)
NS
0
1
2
3
4
5
6
7
Before
After
